# The Association Between Self-perceived Health and Sleep-Quality and Anxiety Among Newly Arrived Refugees in Sweden: A Quantitative Study

**DOI:** 10.1007/s10903-019-00871-z

**Published:** 2019-02-20

**Authors:** Elisabeth Mangrio, Slobodan Zdravkovic, Katarina Sjögren Forss

**Affiliations:** 1 Department of Care Science, Faculty of Health and Society, Malmö University, Malmö, Sweden; 2 Malmö Institute for Studies of Migration, Diversity and Welfare (MIM), Malmö University, Malmö, Sweden

**Keywords:** Migration, Sleep quality, Self-perceived health

## Abstract

Previous research findings suggest that insomnia could be related to decreased health status and that it could also be affected by traumatic life experiences, such as war. Good health is important for newly arrived refugees for an effective integration process. The aim of the present study is, therefore, to investigate the association between self-perceived health and sleep quality among newly arrived refugees in Sweden. The results are based on 681 migrants who participated in a survey between 2015 and 2016. There was a significant odds ratio (OR) after adjustment for confounders for newly arrived refugees that were experiencing bad self-perceived health to also experience bad sleep: OR 8.07 (4.34–15.00). Furthermore, the OR remained significant but lower after adjustments for confounders for newly arrived refugees that had bad self-perceived health to be suffering from anxiety during sleep, with OR 3.83 (2.11–6.94).

## Background

Research findings have shown that 41% of refugees suffer from insomnia, with the symptoms starting at the time they fled from war [1]. Insomnia, that is associated with several health risks, both physical and mental [2], has been found to be prevalent among older as compared to younger refugees, as well as among those who experienced stress during war or those suffering from nightmares [1]. Furthermore, insomnia has also been shown to be associated with various depressive symptoms and depression is the largest and most consistent risk factor for sleep disturbances [1, 3–5]. A number of risk factors for sleep disturbances have been identified including female sex, medical problems and increasing age [6–12]. Findings from Al-Smadi [13] suggest that lower educational level, as well as unemployment, effects insomnia among refugees and that insomnia itself correlates with decreased health [13]. Psychophysiological factors such as stressful life events, hyperarousal and anxiety are often assumed to play a major role in vulnerability to develop sleep disturbances, in particularly insomnia [14–16]. Research indicates that people subjected to traumatic events such as escaping from a war zone, loss of family, violence and forced displacements suffer from sleep disturbances [17–20].

A good health status is necessary for everyday functioning and the individual’s potential for development [21]. This does not differ for newly arrived refugees, where good health is essential for successful integration in the host country [22]. Migration in itself is not a risk factor for health, since migrants are often comparatively healthy. However, vulnerability to physical, mental and social health problems may be the result from the process and circumstances of migration [22]. Thus, it is crucial to examine how sleep disturbances affects the health of newly arrived refugees in order to determine the support and help required for their well-being. However, to our best knowledge, such research is limited. Therefore, this study aims to investigate the association between self-perceived health and sleep quality among newly arrived refugees in the county of Scania in Sweden.

## Methods

### Participants

The study population was recruited from all eligible adult refugees that participated in the civic and health information between February 2015 and February 2016 [23]. Data collection was conducted through a self-administered questionnaire that included questions about health, level of education, sleep, well-being, social relations, accommodation type, work, and access to health care. The questionnaire that was translated by authorized translators and validated by civic and health communicators. A pilot study was conducted prior to the study.

Approximately 1700 questionnaires were distributed by the civic and health communicators to refugees speaking Dari or Arabic and participating in the mandatory public integration support program in the Scania region of Sweden. In total, 681 questionnaires were returned by post, resulting in a response rate of approximately 39.5%.

### Measures

#### Dependent Variable

Self-perceived health was assessed by the following question: How do you consider your current health-situation to be? The answers were grouped into “good” or “bad.” This question has been used earlier in Swedish public health surveys [24, 25].

#### Independent Variables

Sleep quality were assessed by following question: How do you sleep in general? The answers were grouped into “good” or “bad.” Anxiety during sleep was assessed by the following question: Do you have difficulty sleeping due to anxiety? The answers were grouped into “not at all” or “more than usually.” These two questions on sleep and anxiety were derived from the General Health Questionnaire (GHQ) 12 scale [26].

Further, the educational level was based on years of schooling—divided into low educational level (9 years or less), medium educational level (10–12 years of school), and high educational level (more than 12 years).

Age was measured as a continuous variable, but for descriptive statistics, it was divided into five categories: aged 18–34, 35–44, 45–54, 55–64, and 65–80. Gender was divided into either male or female.

### Analysis

Descriptive statistics were calculated as frequencies and percentages. Logistic regression was used to analyze the association between self-perceived health and sleep quality by calculating the odds ratios and the 95% confidence intervals. Multiple logistic regression was used to adjust the estimated odds ratios (OR) for the influence of confounders such as educational level, age, and gender. Statistical analyses were performed by SPSS version 22.

### Ethical Issues

The present study was approved by the Regional Ethical Committee in Lund, Sweden, Approval Number 2014/285. Before participation in the study, all informants received written information about the current study and had to sign a written consent.

## Results

The study included 681 recently arrived refugees who answered the questionnaire. Of the respondents, 204 were females, 461 were males, and 16 did not specify their gender. There were 307 respondents between the ages of 18 and 34 and 155 between the ages of 35 and 44. A total of 301 respondents had a high educational level, 141 had a medium educational level, and 146 had a low educational level. For descriptive statistics on self-perceived health, sleep quality and anxiety, see Table 1.

**Table 1:** Descriptive statistics on self-perceived health, sleep quality and anxiety among newly arrived refugees

	Men N (%)	Women N (%)	All N (%)
Self-percieved health			
Yes	328 (71.1)	128 (62.7)	466 (68.4)
No	131 (28.4)	72 (35.3)	205 (30.1)
Sleep quality			
Good	221 (48)	119 (58.4)	345 (50.7)
Bad	237 (51.5)	85 (41.7)	327 (48)
Anxiety during sleep			
Not at all	269 (58.3)	132 (64.7)	403 (59.2)
More than usually	166 (36)	58 (28.9)	228 (33.5)

Regarding the association between self-perceived health and sleep, there was a significant OR after adjustment for confounders for those experiencing both bad self-perceived health and bad sleep; for this group, the OR was 8.07 (4.34–15.00), as shown in Table 2. The OR was also significant for men and women separately, although higher for men, also shown in Table 2.

**Table 2:** The association between self-perceived health and sleep

Sleep habits	Males		Females		All	
	Number	OR 95% (CI) P-value	Number	OR 95%(CI) P-value	Number	OR 95% (CI) P-value
Good sleep		1		1		1
Bad sleep	209	8.61 (4.12–18.00)	79	6.52 (2.02–21.03)	288	8.07 (4.34–15.00)

Regarding the association between self-perceived health and anxiety during sleep, there was a significant OR after adjustments for confounders for those having bad self-perceived health and suffering from anxiety during sleep—with OR of 3.83 (2.11–6.94), see Table 3. Table 3 also shows that the OR was only significant for men when analyzed separately.

**Table 3:** The association between self-perceived health and experience of anxiety during sleep

Sleep and anxiety	Males		Females		All	
	Number	OR 95%(CI) P-value	Number	OR 95%(CI) P-value	Number	OR 95%(CI) P-value
Not disturbed by anxiety during sleep		1		1		1
Disturbed by anxiety during sleep	209	4.33 (2.08–8.99)	79	2.77 (0.94–8.13)	288	3.83 (2.11–6.94)

## Discussion

The results show that among newly arrived refugees, there is a significant association between self-perceived health and sleeping quality as well as between self-perceived health and anxiety during the night. This is in line with previous research [1, 13]. The associations seen between self-perceived health and sleeping quality and anxiety during sleep in the current study could be explained by different factors. We know that newly arrived refugees are more prone to have experienced stressful life events through them coming from a war-zone [27] and we also know that stressful life events increase the odds for these people to suffer from sleep-disturbances [14–16], which could be an explanation why we also saw these associations in the current study. We know through previous research that insomnia is associated with significant consequences for health, quality of life, social and occupational functions, economics, and public safety [28–31]. Furthermore, insomnia increases work absenteeism and decreases ability to concentrate during work [29, 32]. We also know that newly arrived refugees have to participate in the mandatory public integration support program, which requires energy and concentration [23]. Therefore, it is of great importance to discern and work against factors that could prevent them from fully being able to integrate themselves.

One key actor in this work is the health care professionals who meet these refugees and assist them in different situations [13]. Consequently, it is essential that they are well aware that sleep-disturbance is a common health problem among newly arrived refugees in order for them to detect the symptoms of sleep-disturbance as well as ask about sleeping quality and then help and support those in need. Since sleep-disturbance can be seen as a sign of mental illness, seeking help can be problematic for many individuals due to the stigma associated with it; it is therefore especially important to highlight this issue. By discovering sleep-disturbance at an early stage, serious mental health problems can be prevented—which means health benefits both for the individual and for the society.

The strength of the current study is that it targets health issues highly relevant to the arrival of new refugees to Sweden and the current study was conducted closely after the newly arrived refugees had received permission to stay, which meant that they had been in Sweden approximately between a few months-2 years. As for the limitation, compared to the latest regional public health survey in Scania, the current response rate is low [24]. However, when comparing the response rate of both studies in terms of respondents with a country of birth outside of Europe, the current response rate of 39.5% is moderately higher. Furthermore, the response rate is about the same or higher than that of other studies with similar populations [24, 33]. The response rate resulted from 681 study participants, which is probably why the gender specific analyses were non-significant for women regarding the association between self-perceived health and anxiety during sleep. It could have been beneficial for the study if the population was larger, especially concerning women. The question on self-perceived health has been used in several earlier public health reports and is therefore a well-used question in these kind of surveys in Sweden [25]. The questions on sleep and anxiety that were used as independent variables were derived from the GHQ-12 scale and these questions have shown themselves to be validated when assessed together with the rest of the GHQ-12 questions [26, 34]. Analyzing them as individual questions could be seen as a limitation in this current study as well as there are no sleep-diaries taken and analyzed and this could have been beneficial if it was added into this analysis. However these questions used, have been used in health surveys before and therefore we assume that they are measuring what they intend to measure [24]. We are also aware that the newly arrived refugees could be more or less affected by traumatic life experiences and our inability to not screen for that could also be seen as a limitation. It could be argued that more variables could be added into the adjustment-model for the analysis and we tested for adding Body Mass Index (BMI) but saw that it didn`t change the odds and neither the significance of the results and decided to keep the adjustment-model as it is.
